# Comparative analysis of breeding patterns and reproductive efficiency of mares in subtropical conditions of Pakistan

**DOI:** 10.1002/vms3.1582

**Published:** 2024-08-12

**Authors:** Khalid Mahmood, Mubbashar Hassan, Aijaz Ali Channa, Aamir Ghafoor, Amjad Riaz

**Affiliations:** ^1^ Department of Theriogenology University of Veterinary and Animal Sciences Lahore Punjab Pakistan; ^2^ Department of Clinical Sciences College of Veterinary and Animal Sciences (Sub‐Campus UVAS, Lahore) Lahore Punjab Pakistan; ^3^ University Diagnostic Lab (UDL) at Institute of Microbiology University of Veterinary and Animal Sciences Lahore Punjab Pakistan

**Keywords:** equine reproduction, fertility in mares, follicular dynamics, mares in subtropical climates, oestrus cycle in mares, seasonal breeding

## Abstract

**Background and Aim:**

The present study aimed to evaluate and compare the overall and breed‐specific seasonal breeding patterns, fertility rates, cyclicity, and follicular dynamics of Arab, Thoroughbred, and Percheron mares under the subtropical conditions of Pakistan.

**Materials and Methods:**

A retrospective analysis of climatic data and breeding records of eleven breeding studs spanning four years (2020–2023) was made to find out the overall seasonality in the breeding pattern of mares. Fifty mares of each breed (*n* = 150 in total) were scanned by ultrasonography for a calendar year to find the cyclicity pattern and follicular dynamics (follicular growth rate, size of ovulatory follicle, and days from estrus till ovulation).

**Results and Discussion:**

The statistical analysis of breeding records demonstrated a clear pattern of seasonal breeding (*p*< 0.05). The highest monthly foalings were noted in March (247 ± 45.37), and overall breeding activities peaked in Spring season (*p*< 0.05). Breed‐specific results of Arab, Thoroughbred, and Percheron mares revealed that Arab mares maintained stable breeding activity throughout the year, with the highest activity in spring and peak conception rate in winter (56.25% ± 32.78; *p *> 0.05). Thoroughbred mares experienced significant seasonal declines from spring to winter with a peak conception rate in winter (63.89% ± 27.37, *p *> 0.05). Percherons showed the most pronounced seasonal effects, especially with a high fall conception rate (73.04% ± 19.61) and a sharp decrease in winter breeding metrics (*p*< 0.05). Furthermore, Thoroughbred and Percheron mares displayed the most pronounced seasonal effects on the percentage of cyclic mares 77.3% and 56% in winters (*p*< 0.05). Moreover, the follicular dynamics of the three breeds also exhibited significant differences (*p*< 0.05).

**Conclusion:**

The current study concludes that seasonal and breed‐specific variability exists among the reproductive parameters of Arab, Thoroughbred, and Percheron mares in subtropics, necessitating breed‐specific reproductive management measures to maximize mare breeding efficiency.

## Graphical abstract

Mares exhibit seasonal breeding pattern under subtropical conditions of Pakistan and seasonality has the most pronounced effect on Percheron mares as compared to Arab, and Thoroughbred mares in subtropics.

Thoroughbred and Percheron mares displayed the most pronounced seasonal effects on the percentage of cyclic mares in winters (*p *< 0.05) as compared to Arab Mares.

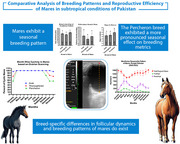

## 1 INTRODUCTION

Equine has played a pivotal role in human history, not only as a source of labour and transportation but also as integral components of cultural, sporting and recreational activities worldwide (Holmes & Brown, 2022). Pakistan is located in the subtropics, and the equine population of the country is enriched with indigenous and exotic breeds having a definite socio‐cultural role (Ashraf et al., 2023; Hassan et al., 2019). Among the exotic breeds, Arab, Thoroughbred and Percheron horses are the most popular in Pakistan. The Arabian horse, deeply rooted in the Middle East and tracing its lineage back over 2000 years in arid landscapes, is known for its endurance, natural beauty, grace and resilience to thrive in harsh conditions (Cosgrove et al., 2020). Conversely, the Thoroughbred, developed under the aegis of the British aristocracy, has been intensely selected over centuries for superior racing capabilities (Bailey et al., 2022; Bower et al., 2012; Hassan et al., 2019). The Percheron breed, originating from France (Al Abri et al., 2020), is primarily utilized for ceremonial and religious purposes in Pakistan.

Mare is a seasonal breeder that has the lowest fertility rate compared to other domesticated species (Sharma, Dhaliwal, et al., 2010), as horse breeds have been selected based on war, draft and racing abilities rather than fertility (Cortés‐Vidauri et al., 2018). To optimize the economic return of broodmares, the number of foals produced per dam lifetime must be maximized (Sharma, Morel, et al., 2010).

The evolutionary perspective shows that horses are best adapted to temperate climates; however, horses are bred invariably in the tropics and subtropics. The seasonality in equine reproduction is significantly influenced by variations in photoperiod, temperature and other climatic conditions (Coelho et al., 2023). The natural breeding season of equine occurs from April to September in the Northern Hemisphere and October to March in the Southern Hemispheres, respectively (Vilhanová et al., 2021).

Environmental factors play a crucial role in determining the reproductive efficiency and overall fertility rates of mares (Nagy et al., 2000; Williams et al., 2012). The environmental signal is translated to an endocrine signal in the pineal gland, which secretes melatonin (Reiter & Sharma, 2021). Melatonin plays an inhibitory role in ovulatory activity via the hypothalamic–pituitary axis (Dowsett et al., 1993; Hamrouni & Benaoun, 2019; Houssou et al., 2021a). The increase in day length activates the hypothalamic–pituitary–ovarian axis in mares by removing the inhibitory effects of melatonin and increasing GnRH production.

Over the last 50 years, seasonal pregnancy rates in mares have risen from 70% to >90% and foaling rates from 55% to >80%. Popular stallions now cover more than 150 mares per season naturally while maintaining high pregnancy rates due to increased precision in determining the timing of ovulation and timed mating with higher pregnancy rates (Allen & Wilsher, 2018). However, studies from subtropics have reported lower fertility rates (Sharma, Dhaliwal, et al., 2010; Sharma, Morel, et al., 2010; Warriach et al., 2014). A study from India concluded that Fertility rates are lower in Thoroughbred mares bred under subtropical climatic conditions than those reported from temperate regions. This might be due to differences in breeding management rather than the prevailing environment (Sharma, Dhaliwal, et al., 2010). Another study from Pakistan reported 52% and 33% conception rates for Arab and Thoroughbred mares at foal heat (Warriach et al., 2014). This study has also noted that the mares remain cyclic throughout the year; however, the conception rates were higher in mares that bred during winter than in summer months. Another study from subtropical conditions has reported that pregnancy rates on foal heat breeding show no significant difference over different months of the breeding seasons despite a rise in temperature (Sharma, Morel, et al., 2010). One study examining climatic influences in the subtropics on the gestational length of Criollo broodmares in the Southern Hemisphere found significant effects of climatic conditions on mare gestation periods (Moraes et al., 2021). Another investigation into the oestrous cycles of Australian Stock Horse mares indicated that oestrus durations were extended during winter and the prevalence of anovulatory oestruses was higher in autumn and winter; however, there was no evidence suggesting that mares undergo two breeding seasons annually (Dowsett et al., 1993).

To maximize pregnancy rates and assist veterinarians in limiting the best period for breeding mares, it is critical to estimate ovulation time with certainty (Hasson & Rahawy, 2022). The optimum time of breeding for a mare in oestrus may be determined by taking into account the changes in uterine oedema, cervical tone, size and shape of ovulatory follicle (McCue, 2021). The size of the ovulatory follicle should be more than 35 mm in mares; however, it varies within and among different breeds (Cortés‐Vidauri et al., 2018; Houssou et al., 2021b). In general, light breeds mares will ovulate a follicle that is smaller in diameter than mares of larger breeds (Ginther et al., 2018). Temperature and rainfall do not affect follicular dynamics, photoperiod has only a small effect on dominant preovulatory follicular diameter, and given the low repeatability of follicular diameter, this characteristic should be evaluated daily to predict ovulation and determine the optimal time to breed the mares (Rua et al., 2019). Once the pre‐ovulatory follicle has reached its maximal size, an ovulation induction therapy is administered to the mares for scheduled breeding purposes (McCue, 2021).

The available data on the reproductive performance of different horse breeds, including oestrus expression and fertility/foaling rates, in the subtropical conditions of Pakistan are currently insufficient. There is a dire need to examine the varied responses of different horse breeds to seasonal fluctuations. There is a significant knowledge gap in determining breed‐specific seasonal patterns, variability in follicular dynamics and identifying the optimal months of the year for horse breeding in subtropical environments.

The objectives of the current study were to evaluate and compare the overall and breed‐specific seasonal breeding patterns, fertility rates, cyclicity patterns and follicular dynamics of Arab, Thoroughbred and Percheron mares under the subtropical conditions of Pakistan.

## 2 MATERIALS AND METHODS

### 2.1 Study location and ethical approval

The study was conducted at 11 different breeding studs in Punjab, Pakistan (30.85° N 72.345° E). All the experiments were approved by the ethical review committee following ARRIVE guidelines (Percie du Sert et al., 2020), and no animal was harmed or euthanized during the study.

### 2.2 Day length and climatic condition

The day length and climatic conditions data were collected from the official websites of timeanddate and weatherspark. The month and season‐wise data of day length and climate were analysed for 4 years (January 2020 to December 2023) to find its impact on breeding pattern of mares under subtropical conditions of Pakistan.

### 2.3 Seasons of the year

Pakistan has four seasons, that is spring, summer, autumn and winter. Based on climate data and previous literature, the months of February and March were included in the spring season, the months from April to September were included in the summer season, the month of October was included in the fall season, and the months of November, December and January were included in winter season (Syed et al., 2021).

### 2.4 Nutrition and housing management

Balanced nutrition (consisting of Oats/Lucerne hay, green fodder, grain‐based concentrate and vegetable oil) and fresh drinking water were provided to all the mares (Hynd, 2019). The brood mares were housed in individual stabling boxes. The mares were dewormed/vaccinated regularly. Good sanitary and hygienic animal housing conditions, feeding mangers and water troughs were ensured regularly. Electric fans were also installed in the animal boxes.

### 2.5 Breeding methods

The mares were given natural covering from selected stallions. All these stallions were selected based on their previous fertility records. Breeding soundness (McCue, 2021) was also evaluated before the start of every breeding season.

### 2.6 Study design

The grouping of the mares was based on ultrasongraphy findings (Neto et al., 2018).

#### 2.6.1 Experiment‐1: seasonality pattern of horse breeding in Pakistan

In this part of the study, a retrospective analysis of breeding data of mares, spanning 4 years (2020–2023), was performed across 11 breeding studs located in Punjab, Pakistan. The purpose of this investigation was to determine the overall seasonality pattern and the best months for equine breeding under the subtropical conditions of Punjab, Pakistan. Thirty‐four hundred and twenty‐nine (*n* = 3429) mares were registered with the 11 well‐established breeding studs at any given month of the year, which were considered to evaluate the overall seasonality pattern. The breeds of the mares registered with these studs were Arab, Thoroughbred, Percheron, the cross of Arab and Thoroughbred (termed Anglo‐Arab) and different local breeds termed desi. The total number of coverings and foalings observed during the study period was 20,657 (*n* = 20,657) and 9970 (*n* = 9970), respectively.

Furthermore, one particular stud (involved in the breeding of pureblood lines of Arab, Thoroughbred and Percheron horses) out of these 11 breeding studs was chosen for breed‐specific analysis. These pureblood mares were born in Pakistan and having a complete pedigree record. This section sought to identify any difference in month‐by‐month and season‐by‐season breeding trends across these three different mare breeds under the subtropical conditions of Punjab, Pakistan. For this breed‐specific study on breeding patterns, there were 269 breedings, 172 foalings and 139 pregnancies for Arab mares; 253 breedings, 169 foalings and 147 pregnancies for Thoroughbred mares; and 253 breedings, 147 foalings and 169 pregnancies for Percheron mares.

#### 2.6.2 Experiment‐2: breed‐specific differences in cyclicity pattern and follicular dynamics of mares in Punjab, Pakistan

One hundred and fifty mares (*n* = 150) of Thoroughbred (*n*_1_ = 50), Arab (*n*_2_ = 50) and Percheron (*n*_3_ = 50) breeds, having a history of no reproductive problems, were selected for this experiment and were followed through visual observation and ultrasonography to determine the cyclicity pattern for a year. The mares included in the cyclicity pattern study were scanned regularly by ultrasonography on every 10th day and grouped into cyclic or acyclic mares on a monthly basis. The grouping of the mares was based on ultrasongraphy findings (Neto et al., 2018). Briefly speaking, mares showing ovarian inactivity, with ovarian follicles less than 15 mm in diameter and no detectable corpus luteum over 3 weeks, were considered mares in anoestrus (acyclic). The mares presenting larger follicles (25–30 mm) but without a corpus luteum over 3 weeks were regarded as transition mares, and the mares that had more than 35 mm follicles along with moderate‐to‐obvious uterine oedema or corpus luteum were considered cyclic mares.

To study the differences in follicular dynamics (follicular growth rate, size of ovulatory follicle and number of days in oestrus till ovulation), ultrasonography was performed on mares that showed visual signs of oestrus to know the ovarian status and uterine oedema. Ultrasonography was performed by using a Draminski iScan2 multi‐ultrasound scanner with a linear rectal probe at 7.0 MHz by a single operator. The mares in oestrus were scanned every 12 h until one day post‐ovulation (Brinsko et al., 2003; Scarlet et al., 2023). The ovarian mapping was performed to know the follicular dynamics (days in oestrus till ovulation, follicular growth rate and the size of pre‐ovulatory follicle).

### 2.7 Study variables

For the study about seasonality patterns (overall and breed‐wise), the independent variables were the month of the year and season of the year. The dependent variables for this study were day length, climatic conditions (temperature and humidity), number of breedings, number of foalings and number of in‐foal mares. For the study about differences in cyclicity pattern, the breed of the mares and month of the year were independent variables, and ovarian status (cyclic or acyclic) and follicular dynamics (days in oestrus till ovulation, follicular growth rate and the size of pre‐ovulatory follicle) of the mares were dependent variables.

### 2.8 Statistical analysis

All the data were checked for normality by Shapiro–Wilk test. To study the differences among day length, climatic conditions (temperature and humidity), number of breedings, number of foalings and number of in‐foal mares due to month of the year and season of the year, one‐way ANOVA at 95% confidence interval was applied. To evaluate the differences in cyclicity pattern (cyclic or acyclic) in three different breeds of the mares due to month of the year and season of the year, a chi‐square test at 95% confidence was applied. To evaluate the effect of the breed of the mares on follicular dynamics (size of ovulatory follicle, growth rate of ovulatory follicle during oestrus and days in oestrus till ovulation), one‐way ANOVA at a 95% confidence interval was applied. All the statistical analyses were performed in SPSS 27.0, and GraphPad Prism 10.0 was used for the graphical illustration of the results.

## 3 RESULTS

### 3.1 Day length

**FIGURE 1:**
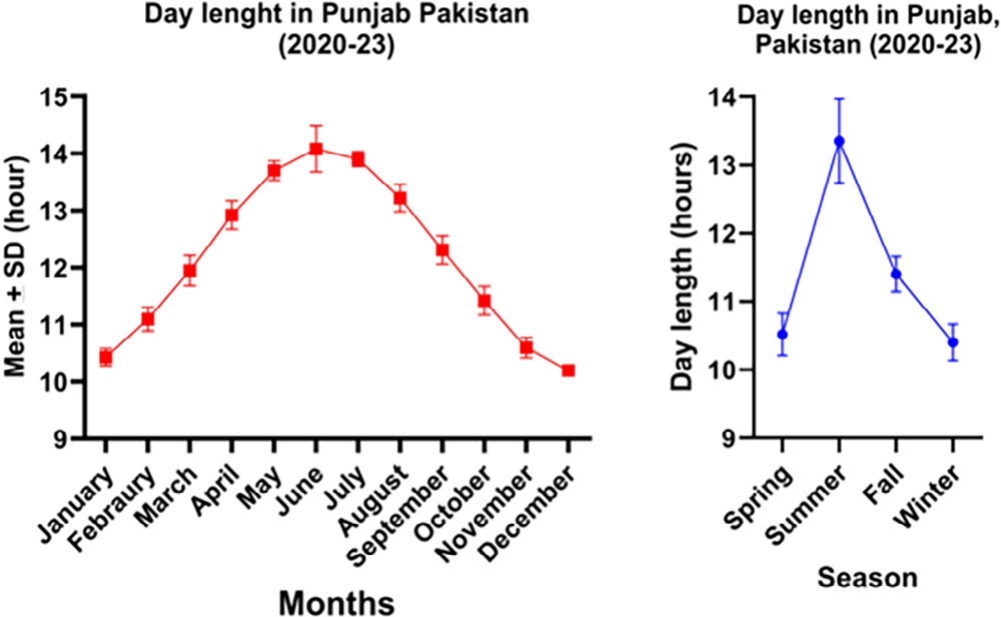
Day length (month‐wise and season‐wise). Detailed results have been provided in Tables S1 and S2.

The analysis of day length data for 4 years (January 2020 to December 2023) revealed a significant difference (*p *< 0.05) in day length among different seasons and months under subtropical conditions of Punjab, Pakistan. Seasonally, day length varied significantly across seasons, with the longest days in summer (average 13:22:17.67 h) and the shortest in winter (average 10:24:36.48 h). Monthly data showed an increase in day length from January (10:25:02.53 h) to June (14:05:50.44 h), then a decrease towards December (10:12:11.56 h), reflecting the broad changes in daylight exposure that animals experience throughout the year. Figure 1 depicts the monthly and seasonal fluctuations in the duration of daylight across Punjab Pakistan, which may impact the breeding cycles of mares.

### 3.2 Climatic condition

**FIGURE 2:**
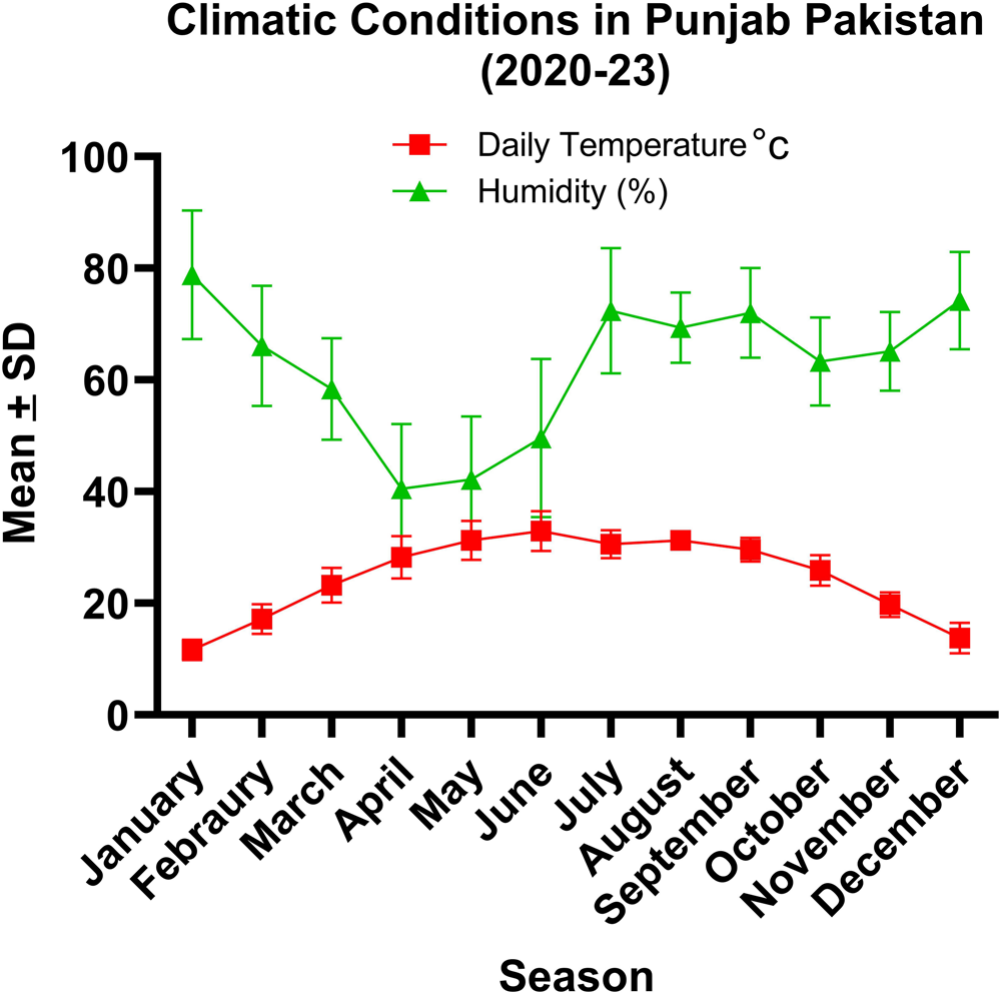
Climatic conditions (daily temperature and humidity).

The analysis of climatic conditions data revealed a clear seasonal trend in temperature and humidity. The highest mean temperature was observed in the summer months, particularly in June, and the lowest in the winter, particularly in December and January. Conversely, humidity levels were highest towards the later part of the year, with peaks often occurring during the monsoon months, indicating a possible inverse relationship between temperature and humidity levels throughout the year. The results are shown in Figure 2.

### 3.3 Seasonality pattern of mares under subtropical conditions of Pakistan

**FIGURE 3:**
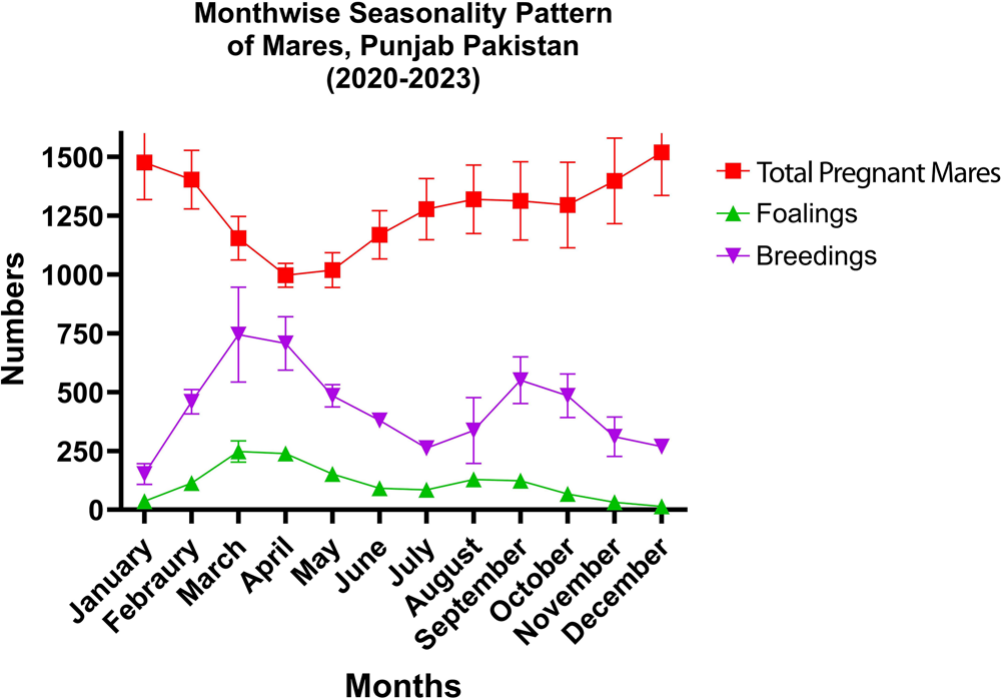
Seasonality pattern of breeding mares under the subtropical condition of Punjab, Pakistan. Detailed results have been provided in Table S3.

The changes in total number of in‐foal mares, foalings and breedings across different months and seasons in Punjab Pakistan were measured. In January, the mean number of in‐foal mares showed the highest value (1476.00 ± 157.04), with a significant decrease observed by April (996.67 ± 51.01). Foaling rates peaked significantly in March (247.33 ± 45.37), whereas the mean numbers of breedings were most frequent in March (744.67 ± 202.24). Notably, each parameter demonstrated significant variability across months (*p *< 0.05), as indicated in Figure 3.

Likewise, spring presented the highest activity in breeding parameters, with a mean number of foalings (180.33 ± 81.15), breedings (601.67 ± 204.90) and in‐foal mares (1279.00 ± 168.27) exhibiting highest values during this season. Conversely, winter showed the lowest foaling rates (27.00 ± 14.02) and a significant reduction in the mean number of breedings (243.22 ± 86.27) and foalings (27.00 ± 14.02). The results have been summarized in Table 1.

**TABLE 1:** Season‐wise overall seasonality pattern in mares under subtropical conditions of Punjab, Pakistan.

Season	Total mares (mean ± SD)	In‐foal (mean ± SD)	Percentage (%) of mares pregnant^1^	Foaling (mean ± SD)	Services (mean ± SD)
Spring	3349 ± 171.84^a^	1279.00 ± 168.27^a^	38.19 ± 4.71^ab^	180.33 ± 81.15^a^	601.67 ± 204.90^a^
Summer	3404 ± 171.19^a^	1182.39 ± 169.50^a^	34.71 ± 4.45^a^	136.22 ± 54.17^ab^	453.06 ± 168.32^a^
Fall	3418 ± 157.07^a^	1295.67 ± 182.54^a^	37.79 ± 3.72^ab^	66.33 ± 7.23^b^	484.33 ± 92.23^a^
Winter	3411 ± 133.91^a^	1464.00 ± 160.17^b^	42.67 ± 4.30^b^	27.00 ± 14.02^bc^	243.22 ± 86.27^b^

*Note*: Values in the column not sharing the same subscript are significantly different at *p* < 0.05.

^1^Means percentage of pregnant mares out of total mares.

### 3.4 Breed‐wise effect of seasonality of fertility parameters of brood mares (Arab, Thoroughbred and Percheron)

The retrospective analysis of breed‐wise effects of seasonality on fertility parameters among broodmares of Arab, Thoroughbred (TBP) and Percheron breeds underlines the significant impact of seasonal changes on reproductive performance. Each breed demonstrated unique responses to seasonal variations in terms of mares bred, mares pregnant, conception rate and mares foaled.

#### 3.4.1 Arab breed

**FIGURE 4:**
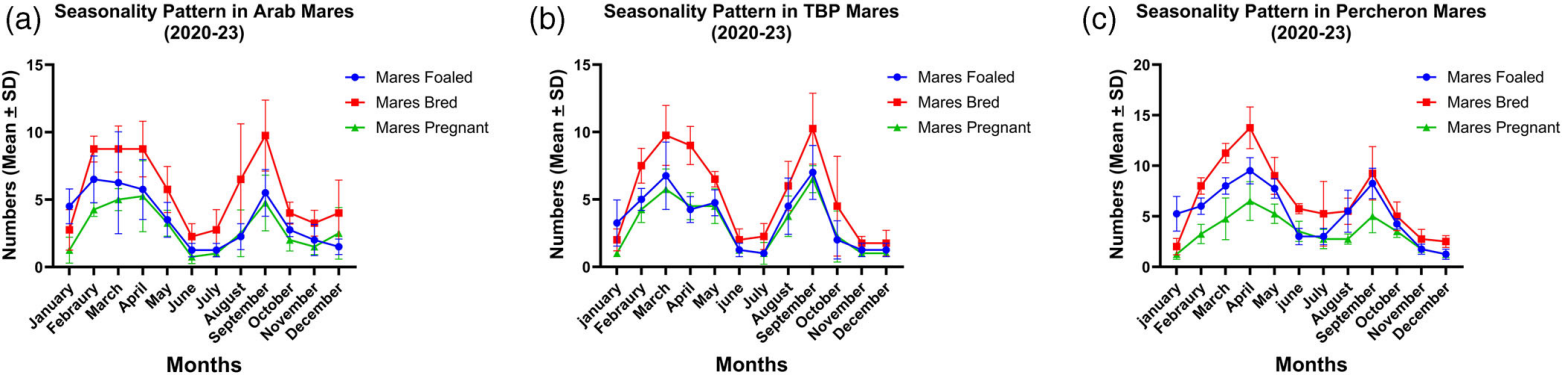
Month‐wise seasonality pattern in Arab (a), TBP (b) and Percheron (c) mares. Detailed in Tables S4–S6.

In the Arab breed, the mean number of mares bred and pregnant decreased from spring to winter, with spring having the highest mean for mares bred (8.75 ± 1.28) and pregnant (4.63 ± 0.74). The conception rate was relatively stable across seasons, peaking in winter (56.25% ± 32.78%), whereas the number of mares foaled was highest in spring (6.38 ± 2.72) and significantly decreased by winter (2.67 ± 1.67). The detailed results are shown in Figure 4 and Table 2.

**TABLE 2:** Season‐wise breeding pattern in Arab mares under subtropical conditions of Punjab, Pakistan (2020–2023).

	Spring (mean ± SD)	Summer (mean ± SD)	Fall (mean ± SD)	Winter (mean ± SD)
Mares bred	8.75 ± 1.28^a^	5.96 ± 3.54^ab^	4.00 ± 0.82^b^	3.33 ± 1.67^bc^
Mares pregnant	4.63 ± 0.74^a^	2.92 ± 2.24^ab^	2.00 ± 0.82^b^	2.00 ± 1.60^bc^
Conception rate (%)	53.25 ± 8.08^a^	47.95 ± 21.67^a^	48.33 ± 11.06^a^	56.25 ± 32.78^a^
Mares foaled	6.38 ± 2.72^a^	3.25 ± 2.23^b^	2.75 ± 0.50^ab^	2.67 ± 1.67^b^

*Note*: Values in the same row not sharing the common superscript are significantly different at *p* < 0.05.

#### 3.4.2 Thoroughbred (TBP) breed

For Thoroughbred mares, similar seasonal trends were observed, with a decrease in mares bred from spring (8.63 ± 2.07) to winter (1.83 ± 0.72). The mares pregnant followed a comparable pattern, highest in spring (5.00 ± 1.41) and lowest in winter (1.00 ± 0.00). The conception rate showed less variability, with the highest rate in winter (63.89% ± 27.37%). Mares foaled also decreased from spring (5.88 ± 1.96) to winter (1.92 ± 1.38). The detailed results are shown in Figure 4 and Table 3.

**TABLE 3:** Season‐wise breeding pattern in Thoroughbred mares under subtropical conditions of Punjab, Pakistan (2020–2023).

	Spring (mean ± SD)	Summer (mean ± SD)	Fall (mean ± SD)	Winter (mean ± SD)
Mares bred	8.63 ± 2.07^a^	6.00 ± 3.44^a^	4.50 ± 3.70^ab^	1.83 ± 0.72^b^
Mares pregnant	5.00 ± 1.41^a^	3.58 ± 2.19^a^	2.25 ± 1.89^a^	1.00 ± 0.00^b^
Conception rate (%)	58.18 ± 10.19^a^	58.33 ± 19.73^a^	50.00 ± 13.61^a^	63.89 ± 27.37^a^
Mares foaled	5.88 ± 1.96^a^	3.79 ± 2.43^ab^	2.00 ± 1.41^b^	1.92 ± 1.38^bc^

*Note*: Values in the same row not sharing the common superscript are significantly different at *p* < 0.05.

#### 3.4.3 Percheron breed

The Percheron breed exhibited a more pronounced seasonal effect, with the number of mares bred significantly higher in spring (9.63 ± 1.92) compared to winter (2.42 ± 0.79). Mares pregnant and foaled followed similar patterns, with notable decreases from spring to winter. Interestingly, the conception rate for Percherons was the highest in fall (73.04% ± 19.61%). The detailed results are shown in Figure 4 and Table 4.

**TABLE 4:** Season‐wise breeding pattern in Percheron mares under subtropical conditions of Punjab, Pakistan (2020–2023).

	Spring (mean ± SD)	Summer (mean ± SD)	Fall (mean ± SD)	Winter (mean ± SD)
Mares bred	9.63 ± 1.92^a^	8.08 ± 3.60^ab^	5.00 ± 1.41^b^	2.42 ± 0.79^c^
Mares pregnant	4.00 ± 1.69^a^	4.29 ± 1.81^a^	3.50 ± 0.58^a^	1.42 ± 0.51^b^
Conception rate (%)	41.05 ± 12.65^ac^	55.35 ± 13.32^ab^	73.04 ± 19.61^bd^	62.4 ± 24.74^cd^
Mares foaled	7.00 ± 1.31^a^	6.17 ± 2.84^ab^	4.25 ± 0.50^bc^	2.75 ± 2.09^c^

*Note*: Values in the same row not sharing the common superscript are significantly different at *p* < 0.05.

### 3.5 Breed‐wise cyclicality percentage (month‐wise and season‐wise)

#### 3.5.1 Month‐wise analysis

**FIGURE 5:**
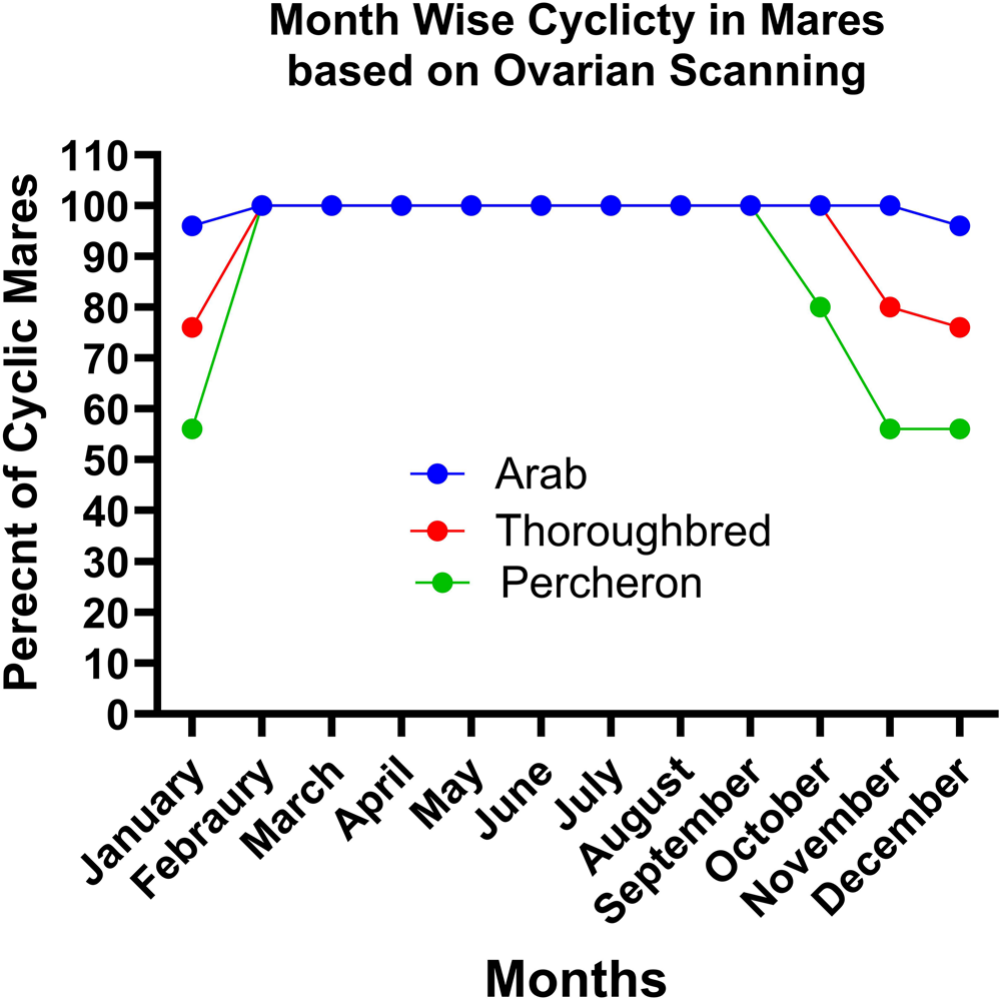
Month‐wise cyclicity pattern of Arab, TBP and Percheron mares. Detailed results have been provided in Table S7.

The percentages of cyclic and acyclic mares were examined across all three breeds from January to December. In January, Arab mares displayed the highest cyclicity at 96% (48/50), with Thoroughbreds at 76% (38/50) and Percherons showing the lowest at 56% (28/50). February marked a period of universal cyclicity across all breeds, with each achieving a 100% (50/50) cyclicity rate, which persisted through to September. A notable decrease in cyclicity was observed in Percherons by October (80%, 40/50) and more significantly in November and December, paralleling the initial trends seen in January, as illustrated in Figure 5.

#### 3.5.2 Season‐wise analysis

When cyclicity percentages were aggregated seasonally, all breeds exhibited optimum cyclicity in spring and summer, indicating optimal reproductive conditions during these seasons. However, a divergence emerged in the fall, with Percherons dropping to 80% (40/50), and further in winter, whereas Arab mares maintained a relatively high cyclicity of 97.3% (146/150), Thoroughbreds reduced to 77.3% (116/150) and Percherons the lowest at 56.0% (84/150). The statistical significance of these variations, especially in winter, highlights breed‐specific adaptability to seasonal changes. The results have been summarized in Table 5.

**TABLE 5:** Season‐wise cyclicity pattern in Arab, TBP and Percheron mares under subtropical conditions of Punjab, Pakistan.

Season	Breed of mares		
	Arab	TBP	Percheron
Spring	100% (100/100)^a^	100% (100/100)^a^	100% (100/100)^a^
Summer	100% (300/300)^a^	100% (300/300)^a^	100% (300/300)^a^
Fall	100% (50/50)^a^	100% (50/50)^a^	80.0% (40/50)^b^
Winter	97.3% (146/150)^a^	77.3% (116/150)^b^	56.0% (84/150)^c^
*p*‐Value	0.162	0.001	0.001

*Note*: Values in the same row not sharing the same subscript are significantly different at *p* < 0.05.

### 3.6 Effect of breed of mare on follicular dynamics and days in oestrus

The average size of the pre‐ovulatory follicle varied significantly among the breeds, with Percherons having the largest follicles (46.16 ± 3.69 mm), followed by Arabs (42.18 ± 3.27 mm) and Thoroughbreds (38.62 ± 2.68 mm). These differences indicate breed‐specific variations in follicular development, with Percherons demonstrating a capacity for ovulation at larger follicular sizes.

Follicular growth rates also differed, with Arabs (3.09 ± 1.69 mm/day) and Percherons (2.91 ± 1.16 mm/day) showing similar rates, which were significantly higher than that of Thoroughbreds (2.02 ± 0.74 mm/day). This suggests that although Arab and Percheron breeds exhibit faster follicular growth, Thoroughbreds have a slower rate of follicular development.

**FIGURE 6:**
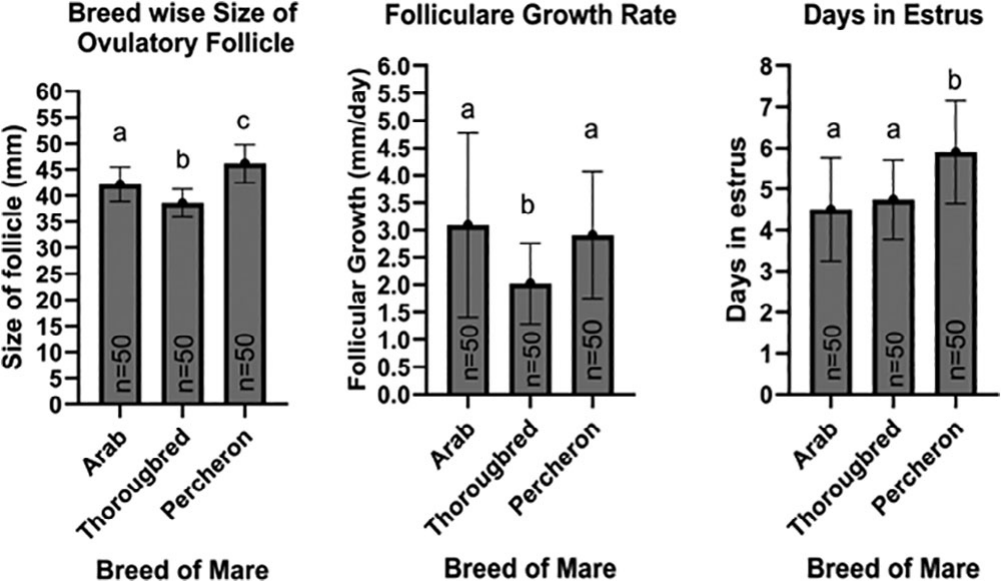
Effect of breed of mare on follicular dynamics and days in oestrus. Detailed in Table S8.

Regarding the duration of oestrus, Percherons exhibited the longest periods of heat until ovulation (5.90 ± 1.25 days), which was significantly longer than both Arab (4.50 ± 1.26 days) and Thoroughbred (4.74 ± 0.96 days) mares. The results have been summarized in Figure 6.

## 4 DISCUSSION

The current study has identified significant breed‐specific differences in reproductive responses of Arab, Thoroughbred and Percheron mares under the subtropical climate of Pakistan, with Arab mares showing high adaptability and Arab and Thoroughbred displaying less variability in conception rates throughout the year; however, Percheron mares experiencing pronounced seasonal fluctuations.

The day length continuously varies over the months and seasons (Warriach et al., 2014), and similar results were obtained in our study; however, the climatic data showed summer stress, especially in July and August, as both the temperature and humidity remained higher during these months. This finding was in accordance with previous findings (Abbas, 2013; Saifullah et al., 2022; Warriach et al., 2014). The overall results of the total number of in‐foal mares, breedings and foalings exhibited a seasonal variation in our study. In January, the total number of in‐foal mares was the highest, and it decreased by April due to consistent foaling. The foaling rates peaked in March, coinciding with the highest frequency of services. Spring exhibited the highest breeding activity. In contrast, winter had the lowest foaling rates and a significant reduction in number of mares bred, likely due to a decrease in daylight length. This was in accordance with previous studies that described breeding season in equine as from spring till the end of summer (Abd El‐Razek & Mahboub, 2019; Ginther et al., 2018; Vilhanová et al., 2021). However, breeding and foaling activities displayed a decreased trend in the peak summer months (June, July and August) in our study. The apparent reason behind this trend was heat stress due to an increase in environmental temperature and humidity. This trend was in accordance with previous studies that have reported a decrease in the reproductive performance of mammals due to heat stress (Campbell, 2014; Shakeel & Yoon, 2023). According to our study, the spring season and the months of March and April were the peak time for the breeding activities of mares under the subtropical conditions of Pakistan. However, our data clearly describe that breeding activities never totally ceased throughout the year and continued during the winter season as well, though at a significantly lower rate (*p* < 0.05). It is pertinent to mention here that the overall reproductive performance of mares (fairly above 50%) in our study was lower than the latest reports from developed countries (Allen & Wilsher, 2018) and warrants a detailed overview of breeding management practices to suggest a way to improve it. Another study from Pakistan has reported a 22%–66% conception rate per mating for Thoroughbred mares and a 52%–75% conception rate per mating for Arab mares (Warriach et al., 2014). Our recent study has reported an embryo recovery rate of 68.9% with timed artificial insemination in polo mares (Mahmood et al., 2024), indicating a possible scope of improvement in the equine breeding industry of subtropical countries like Pakistan.

Seasonality effects on fertility parameters among Arab, Thoroughbred and Percheron broodmares demonstrated a pronounced decrease in breeding activities from spring to winter but with notable breed‐specific variations. The Arab and Thoroughbred breeds showed similar patterns, with both reaching peak numbers of mares bred and pregnant in spring, followed by significant declines by winter; however, Arabs maintained a relatively stable conception rate throughout the year, peaking in winter, whereas Thoroughbreds exhibited less variability but their highest conception rate in winter as well. This was in accordance with a previous study from Pakistan, which reported a higher conception rate in Arab mares during winter (Warriach et al., 2014). Percherons, in contrast, showed the most dramatic seasonal effects, with the highest breeding numbers in spring and significantly lower figures in winter. However, their peak conception rate occurred in the fall, indicating a unique breed‐specific breeding pattern. Overall, although all breeds had lower breeding activity in winter, which was in accordance with previous studies from subtropical countries (Sharma, Dhaliwal, et al., 2010; Sharma, Morel, et al., 2010; Warriach et al., 2014), Arab mares have not exhibited a reduced reproductive cyclicity trend in our study, suggesting an adaptation to the subtropical environment.

In the investigation of month‐ and season‐wise cyclicity percentages, distinct patterns emerged across the breeds throughout the year. Seasonally, cyclicity was consistent for all breeds during spring and summer, demonstrating optimal reproductive conditions. In the fall, Percherons showed a decrease in cyclicity to 80%, and this divergence became more pronounced in the winter. During this colder season, Arabs maintained a high cyclicity at 97.3%, whereas Thoroughbreds and Percherons displayed significantly lower rates, 77.3% and 56.0%, respectively. The statistical analysis confirmed significant differences among the breeds in winter, underscoring breed‐specific adaptability to seasonal variations. This variability, particularly notable in Percherons during the fall and winter, suggests a need for tailored management practices to optimize breeding outcomes for different horse breeds under subtropical conditions.

Follicular dynamics and oestrus duration till ovulation across different breeds of mares showed significant variations (*p *< 0.05), suggesting breed‐specific reproductive traits. Percherons displayed the largest pre‐ovulatory follicle (46.16 ± 3.69 mm), significantly larger than that observed in Arab (42.18 ± 3.27 mm) and Thoroughbred (38.62 ± 2.68 mm) mares. This indicates that Percheron mares may have a genetic predisposition towards larger follicular dimensions, which must be considered when predicting timing ovulation in Percheron mares. All these results were in accordance with previous studies which have reported larger ovulatory follicle size in draft mares (Hasson & Rahawy, 2022; McCue, 2021). The follicular growth rates, both Arab and Percheron breeds exhibited comparable and higher rates of follicular enlargement, with means of 3.09 ± 1.69 and 2.91 ± 1.16 mm/day, respectively, whereas Thoroughbred mares exhibited a significantly slower growth rate of 2.02 ± 0.74 mm/day. However, all these values were in accordance with previous studies, which have reported 2–4 mm/day follicular growth in mares during the oestrus phase (Houssou et al., 2021b).

The time taken from the start of oestrus till ovulation showed Percherons experiencing significantly longer periods at 5.90 ± 1.25 days, compared to 4.50 ± 1.26 days in Arabs and 4.74 ± 0.96 days in Thoroughbreds. These findings highlight distinct reproductive cycles among the breeds, with Percherons having notably extended oestrus compared to the other breeds, which could influence their breeding management. Overall, the observed breed differences in follicle size, growth rate and oestrus duration are critical for understanding and managing breed‐specific timed breeding programmes. This study can help mare breeders to manage breeding programmes in a better way to improve the mare reproduction rate with a reduced burden on stallions under subtropical conditions.

## 5 CONCLUSION

It can be inferred from the current study that seasonality has a pronounced effect on Percheron mares compared to Arab and Thoroughbred mares in subtropical regions. Furthermore, breed‐specific differences in mares’ follicular dynamics and breeding patterns should be considered for timed breeding and optimum fertility outcomes.

## AUTHOR CONTRIBUTIONS

**Khalid Mahmood**: Conceptualization; methodology; data curation; statistical analysis; writing—review and editing. **Mubbashar Hassan**: Methodology; writing—review and editing; validation. **Aijaz Ali Channa**: Methodology; writing—review and editing. **Amir Ghafoor**: Writing—review and editing. **Amjad Riaz**: Conceptualization; writing—review and editing; validation; supervision.

## CONFLICT OF INTEREST STATEMENT

The authors declare no conflicts of interest that might affect the impartiality of this article.

## FUNDING INFORMATION

None.

### ETHICS STATEMENT

All the experiments were approved by the ethical review committee of the University of Veterinary and Animal Sciences Lahore (DR/775 dated 20 Feb 2021), following ARRIVE guidelines (Percie du Sert et al., 2020), and no animal was harmed or euthanized during the study.

### PEER REVIEW

The peer review history for this article is available at https://publons.com/publon/10.1002/vms3.1582.

## Supporting information

Supporting Information

## Data Availability

The data that support the findings of this study are available from the corresponding author upon reasonable request.
