# Supplementary material for: Comparative analysis of breeding patterns and reproductive efficiency of mares in subtropical conditions of Pakistan
Source: Vet Med Sci. 2024 Aug 12;10(5):e1582. doi: 10.1002/vms3.1582 (PMC11317926; doi:10.1002/vms3.1582)
Supplement: Supplementary file 1 — Supporting Information [file VMS3-10-e1582-s001.docx]

**SUPPLEMENTARY INFORMATION**

**Table S1: Month-wise Day length under subtropical**

**conditions of Punjab, Pakistan (2020-23)**

| **Monthwise Wise Day Length** | | |
| --- | --- | --- |
| **Month** | **Unit** | **(Mean** + **SD)** |
| Jan | Hours | 10:25:02.53 + 0:09:16.65^a^ |
| Feb | Hours | 11:06:19.77+ 0:13:50.85 ^b^ |
| March | Hours | 11:59:41.98 + 0:16:45.41 ^c^ |
| April | Hours | 12:55:28.74 + 0:15:15.43 ^d^ |
| May | Hours | 13:42:20.46 + 0:11:17.95 ^e^ |
| June | Hours | 14:05:50.44 + 0:02:20.50 ^f^ |
| July | Hours | 13:54:21.62 + 0:08:26.25 ^g^ |
| August | Hours | 13:13:43.22+ 0:14:30.29 ^h^ |
| September | Hours | 12:19:43.64+ 0:15:29.63 ^i^ |
| October | Hours | 11:25:10.40 + 0:15:53.40 ^j^ |
| November | Hours | 10:36:59.31 + 0:11:28.30 ^k^ |
| December | Hours | 10:12:11.56 + 0:02:38.93 ^l^ |

**Note***: Values in the same row not sharing the same column and not sharing common superscript are significantly different at p< .05*

**Table S2: Season-wise Day Length under subtropical conditions of Punjab Pakistan (2020-23)**

| **Season Spring** | | **Summer** | **Fall** | **Winter** |
| --- | --- | --- | --- | --- |
| **Day length** | 11:34:15.19+0:30:50.21 ^a^ | 13:22:17.67+0:38:11.47 ^b^ | 11:25:10.40+0:15:53.40 ^c^ | 10:24:36.48+0:13:16.29 ^d^ |

| **Table S3: Month-wise Seasonality Pattern in Mares under subtropical conditions of Punjab Pakistan** | | | | | |
| --- | --- | --- | --- | --- | --- |
| **Month** | **Parameters** | | | | |
|  | **Total Mares**  **(Mean + SD)** | **Infoal Mares**  **(Mean + SD)** | **%age of Infoal Mares (Mean + SD)^1^** | **Foaling**  **(Mean + SD)** | **Services**  **(Mean + SD)** |
| January | 3344.33+173.48 ^a^ | 1476.00+157.04^a^ | 44.13+3.70 | 36.33+8.74^ah^ | 151.67+43.94^a^ |
| February | 3339.67+179.92 ^a^ | 1403.67+124.81 ^ab^ | 42.04+2.98 | 113.33+30.62^bdg^ | 458.67+52.08^bcdehi^ |
| March | 3360.00+202.84 ^a^ | 1154.33+92.64 ^ab^ | 34.35+1.51 | 247.33+45.37^c^ | 744.67+202.24^b^ |
| April | 3394.33+223.33 ^a^ | 996.67+51.01 ^b^ | 29.38+0.50 | 238.67+27.43^c^ | 706.67+113.32^b^ |
| May | 3405.67+221.62 ^a^ | 1018.67+73.42 ^bc^ | 29.95+2.04 | 151.33+5.69^b^ | 484.00+47.13^bcdehi^ |
| June | 3422.00+205.87 ^a^ | 1168.67+102.16 ^ab^ | 34.16+2.34 | 91.33+2.08^ab^ | 379.33+26.41^acfg^ |
| July | 3407.67+208.98 ^a^ | 1277.33+130.25 ^ab^ | 37.44+2.35 | 84.67+15.31^adef^ | 261.33+9.29^adfg^ |
| August | 3397.33+177.24 ^a^ | 1320.00+145.62 ^ab^ | 38.79+2.79 | 128.00+18.19^beg^ | 336.67+139.55^aefg^ |
| September | 3399.67+178.64 ^a^ | 1313.00+166.95 ^ab^ | 38.52+3.04 | 123.33+12.06^bfg^ | 550.33+98.86^bfhi^ |
| October | 3418.67+157.07 ^a^ | 1295.67+182.54 ^ab^ | 37.74+3.72 | 66.33+7.23^agh^ | 484.33+92.23^bghi^ |
| November | 3413.00+165.81 ^a^ | 1397.33+182.24 ^ab^ | 40.24+4.69 | 31.00+16.37^ah^ | 309.67+83.92^ah^ |
| December | 3477.00+30.61 ^a^ | 1518.67+182.72 ^a^ | 43.65+4.92 | 13.67+4.51^h^ | 268.33+25.54^ai^ |

*Note: Values in the same column and not sharing common superscript are significantly different at p< .05.* ***^1^****Means, percentage of pregnant mares out of total mares.*

| **Table S4: Month-wise Breeding Pattern in Arab Mares under subtropical conditions of Punjab Pakistan (2020-23)** |
| --- |

| **Serial** | **Month** | **n** | **Mares Foaled** | **Mares Bred** | **Mares**  **Pregnant** | **Conception Percentage** |
| --- | --- | --- | --- | --- | --- | --- |
| **1** | January | 4 | 4.50+1.29^a^ | 2.75+1.50 ^ac^ | 1.25+0.96 ^ab^ | 37.50+25.00^a^ |
| **2** | Feb | 4 | 6.50+1.73 ^a^ | 8.75+0.95 ^b^ | 4.25+0.50 ^a^ | 48.61+2.78 ^a^ |
| **3** | March | 4 | 6.25+3.77 ^a^ | 8.75+1.71 ^ab^ | 5.00+0.82 ^a^ | 57.89+9.35 ^a^ |
| **4** | April | 4 | 5.75+2.22 ^a^ | 8.75+2.06 ^abc^ | 5.25+2.63 ^ab^ | 57.11+16.50 ^a^ |
| **5** | May | 4 | 3.50+1.29 ^a^ | 5.75+1.70 ^abc^ | 3.25+0.96 ^ab^ | 56.67+8.17 ^a^ |
| **6** | June | 4 | 1.25+0.50 ^a^ | 2.25+0.96 ^c^ | 0.75+0.50 ^b^ | 29.17+20.97 ^a^ |
| **7** | July | 4 | 1.25+0.50 ^a^ | 2.75+1.50 ^ac^ | 1.00+0.00 ^bc^ | 50.00+35.36 ^a^ |
| **8** | August | 4 | 2.25+0.96 ^a^ | 6.50+4.12 ^abc^ | 2.50+1.73 ^ab^ | 42.50+15.00 |
| **9** | Sep | 4 | 5.50+1.73 ^a^ | 9.75+2.63 ^abc^ | 4.75+2.06 ^ab^ | 52.23+24.33 ^a^ |
| **10** | Oct | 4 | 2.75+0.50 ^a^ | 4.00+0.81 ^ac^ | 2.00+0.81 ^ab^ | 48.33+11.06 ^a^ |
| **11** | November | 4 | 2.00+1.15 ^a^ | 3.25+0.96 ^ac^ | 1.50+0.58 ^bd^ | 45.83+8.34 ^a^ |
| **12** | December | 4 | 1.50+0.58 ^a^ | 4.00+2.45 ^abc^ | 2.50+1.91 ^ab^ | 66.67+30.43 ^a^ |

| Note: Values in the same column and not sharing the same subscript are significantly different at *p< .05* |
| --- |

| **Table S5: Month-wise Breeding Pattern in Thoroughbred Mares under subtropical conditions of Punjab Pakistan (2020-23)** |
| --- |

| **Serial** | **Month** | **n** | **Mares Foaled** | **Mares Bred** | **Mares Pregnant** | **Conception Percentage** |
| --- | --- | --- | --- | --- | --- | --- |
| **1** | January | 4 | 3.25+1.71 ^abc^ | 2.00+0.82 ^ad^ | 1.00+0.00 ^a^ | 58.33+28.87^a^ |
| **2** | Feb | 4 | 5.00+0.82 ^a^ | 7.50+1.29 ^b^ | 4.25+0.96 ^bc^ | 56.90+10.42 ^a^ |
| **3** | March | 4 | 6.75+2.50 ^abc^ | 9.75+2.22 ^bc^ | 5.75+1.50 ^bc^ | 59.46+11.37 ^a^ |
| **4** | April | 4 | 4.25+0.96 ^ab^ | 9.00+1.41 ^b^ | 4.50+1.00 ^bc^ | 49.75+4.14 ^a^ |
| **5** | May | 4 | 4.75+0.96 ^a^ | 6.50+0.58 ^b^ | 4.50+1.29 ^abc^ | 68.45+14.72 ^a^ |
| **6** | June | 4 | 1.25+0.50 ^bc^ | 2.00+0.82 ^ad^ | 1.25+0.50 ^ab^ | 66.67+23.57 ^a^ |
| **7** | July | 4 | 1.00+0.00 ^c^ | 2.25+0.96 ^acd^ | 1.00+0.82 ^ab^ | 37.50+28.47 ^a^ |
| **8** | August | 4 | 4.50+2.08 ^abc^ | 6.00+1.82 ^abd^ | 3.75+1.50 ^abc^ | 62.23+15.73 ^a^ |
| **9** | Sep | 4 | 7.00+2.00 ^abc^ | 10.25+2.63 ^ab^ | 6.50+1.00 ^c^ | 65.39+12.06 ^a^ |
| **10** | Oct | 4 | 2.00+1.41 ^abc^ | 4.50+3.70 ^abd^ | 2.25+1.89 ^abc^ | 50.00+13.61 ^a^ |
| **11** | November | 4 | 1.25+0.50 ^bcd^ | 1.75+0.50 ^d^ | 1.00+0.00 ^a^ | 62.50+25.00 ^a^ |
| **12** | December | 4 | 1.25+0.50 ^bce^ | 1.75+0.96 ^ad^ | 1.00+0.00 ^a^ | 70.83+34.36 ^a^ |

| Note: Values in the same column and not sharing the same subscript are significantly different at *p< .05* |
| --- |

| **Table S6: Month-wise Breeding Pattern in Percheron Mares under subtropical conditions of Punjab Pakistan (2020-23)** |
| --- |

| **Serial** | **Month** | **n** | **Mares Foaled** | **Mares Bred** | **Mares Pregnant** | **Conception Percentage** |
| --- | --- | --- | --- | --- | --- | --- |
| **1** | January | 4 | 5.25+1.71 ^abe^ | 2.00+0.81 ^a^ | 1.25+0.50 ^a^ | 70.83+34.36^a^ |
| **2** | Feb | 4 | 6.00+0.82 ^ab^ | 8.00+0.82 ^bc^ | 3.25+0.96 ^ab^ | 40.13+9.25 ^a^ |
| **3** | March | 4 | 8.00+0.82^a^ | 11.25+0.96 ^b^ | 4.75+2.06 ^ab^ | 41.97+16.90 ^a^ |
| **4** | April | 4 | 9.50+1.29^a^ | 13.75+2.06^b^ | 6.50+1.91 ^ab^ | 46.85+8.65 ^a^ |
| **5** | May | 4 | 7.75+0.96 ^a^ | 9.00+1.82 ^bcf^ | 5.25+0.95 ^b^ | 58.55+3.45 ^a^ |
| **6** | June | 4 | 3.00+0.82 ^be^ | 5.75+0.50 ^cf^ | 3.50+1.00 ^ab^ | 61.67+19.91 ^a^ |
| **7** | July | 4 | 3.00+0.82 ^bce^ | 5.25+3.20 ^abc^ | 2.75+0.95 ^ab^ | 57.92+15.83 ^a^ |
| **8** | August | 4 | 5.50+2.08 ^abe^ | 5.50+1.29 ^acd^ | 2.75+0.50 ^ab^ | 53.39+19.48 ^a^ |
| **9** | Sep | 4 | 8.25+1.50 ^ab^ | 9.25+2.63 ^abc^ | 5.00+1.63 ^ab^ | 53.69+8.13 ^a^ |
| **10** | Oct | 4 | 4.25+0.50 ^bd^ | 5.00+1.41 ^ace^ | 3.50+0.57 ^ab^ | 73.04+19.61 ^a^ |
| **11** | November | 4 | 1.75+0.50 ^e^ | 2.75+0.96 ^af^ | 1.75+0.50 ^a^ | 66.67+23.57 ^a^ |
| **12** | December | 4 | 1.25+0.50 ^ef^ | 2.50+0.58 ^a^ | 1.25+0.50 ^a^ | 49.83+13.34 ^a^ |

| Note: Values in the same column and not sharing the same subscript are significantly different at *p< .05* |
| --- |

| **Table S7: Month-wise Cyclicity Pattern in Arab, TBP and Percheron Mares under subtropical conditions of Punjab Pakistan** | | | |
| --- | --- | --- | --- |
| **Month** | **Breed of Mares** | | |
|  | **Arab** | **TB** | **Percheron** |
| **Jan** | 96% (48/50) ^a^ | 76% (38/50) ^b^ | 56.0% (28/50) ^b^ |
| **Feb** | 100% (50/50) ^a^ | 100% (50/50) ^a^ | 100% (50/50) ^a^ |
| **March** | 100% (50/50) ^a^ | 100% (50/50) ^a^ | 100% (50/50) ^a^ |
| **April** | 100% (50/50) ^a^ | 100% (50/50) ^a^ | 100% (50/50) ^a^ |
| **May** | 100% (50/50) ^a^ | 100% (50/50) ^a^ | 100% (50/50) ^a^ |
| **June** | 100% (50/50) ^a^ | 100% (50/50) ^a^ | 100% (50/50) ^a^ |
| **July** | 100% (50/50) ^a^ | 100% (50/50) ^a^ | 100% (50/50) ^a^ |
| **August** | 100% (50/50) ^a^ | 100% (50/50) ^a^ | 100% (50/50) ^a^ |
| **September** | 100% (50/50) ^a^ | 100% (50/50) ^a^ | 100% (50/50) ^a^ |
| **October** | 100% (50/50) ^a^ | 100% (50/50) ^a^ | 80% (40/50) ^ab^ |
| **November** | 100% (50/50) ^a^ | 80% (40/50) ^ab^ | 56.0% (28/50) ^b^ |
| **December** | 96% (48/50) ^a^ | 76% (38/50) ^b^ | 56.0% (28/50) ^b^ |
| ***P*-Value** | 1.000 | **0.001** | **0.001** |

*Note: Values in the same row not sharing the same subscript are significantly different at p< .05*

| **Table S8: Effect of Breed of Mare on Follicular dynamics and days in estrus** | | | |
| --- | --- | --- | --- |
| **Parameters** | **Breed** | | |
|  | **Arab** | **TBP** | **Percheron** |
| **Size of the follicle (mm)** | 42.18+3.27^a^ | 38.62+2.68 ^b^ | 46.16+3.69 ^c^ |
| **Follicular Growth per day (mm)** | 3.09+1.69 ^a^ | 2.02+0.74 ^b^ | 2.91+1.16 ^a^ |
| **Days in heat till Ovulation** | 4.50+1.26 ^a^ | 4.74+0.96 ^a^ | 5.90+1.25 ^b^ |
| *Note: Values in the same row not sharing the common subscript are significantly different at p< .05* | | | |
